# Correction to “A portrait of Endoscopic retrograde cholangiopancreatography and endoscopic ultrasound training programs in Europe: Current practices and opportunities for improvement”

**DOI:** 10.1002/ueg2.12434

**Published:** 2023-06-30

An incomplete version of Table 3 was published in this article. The updated version of this table appears below.

**TABLE 3:** Adherence rate to ESGE Recommendations on ERCP/EUS Training.

Number of ESGE recommendation	Number of positive answers	Number of negative answers	% adherence to the recommendation
1. Every endoscopist should have achieved competence in UGI endoscopy before commencing training in ERCP or EUS, that is, having personal experience of at least 300 gastroscopies and meeting the ESGE quality measures for UGI endoscopy	24	6	80%
2a. Simulation‐based training represents a positive development to accelerate the trainee's learning curve and should be encouraged.	22	19	53.7%
2b, 3. When available, trainees should start training by undertaking structured supervised ERCP/EUS simulator‐based training before commencing hands‐on training in the workplace. Where it is available, simulation‐based training should evolve in a stepwise approach for training: Virtual reality and mechanical simulators should be used during early training, followed by hands‐on endoscopy training	10	12	45.5%
4, 5. Trainees should undertake formal courses to complement ERCP/EUS training. ERCP and EUS trainees should engage with a range of learning resources to supplement formal courses and experiential learning.	14	8	63.6%
6. ERCP and EUS training should follow a structured syllabus to guide what is covered in workplace learning, formal training courses, and self‐directed study	22	19	53.7%
7. A minimum training period of 12 months of high volume training is likely to be required to obtain minimum proficiency in both ERCP and diagnostic EUS.	22	19	53.7%
8. A significant proportion of training should be based in high volume* training centers that are able to offer trainees sufficient wealth of experience for at least 12 months:			
8a. ERCP	39	2	95.1%
8b. EUS	37	4	90.2%
9. An ERCP/EUS training center should ideally be able to provide:			
9a. Multidisciplinary hepatobiliopancreatic meetings	37	4	90.2%
9b. Onsite hepaticopancreaticobiliary surgery	37	4	90.2%
9c. Onsite interventional radiology	39	2	95.1%
9d. ERCP and EUS simulations	21	20	51.2%
9e. Involvement in research and service improvement initiatives	36	5	87.8%
10, 11. A trainee's principal trainer should ideally have more than 3 years of experience of independent ERCP and/or EUS practice.	33	8	80.5%
13. Formal assessment tools should be used regularly during ERCP and EUS training to track the acquisition of trainees' competence and to support trainee feedback	7	20	25.9%
14. Trainees should be encouraged to undertake self‐assessment and keep a contemporaneous logbook of all cases, which includes the degree of trainer support that was needed for each aspect of the procedure	11	19	36.7%
15. A trainee should undergo:			
15a. Formal summative assessment process	27	14	65.9%
15b. Prior to commencing independent practice in ERCP/EUS	8	19	29.6%
20, 28. The number of ERCP/EUS performed may be a surrogate marker of competence, but in isolation is an inexact means to demonstrate competence. Most trainees are likely to need to have performed >300 ERCPs/>250 diagnostic EUSs to be in a position to demonstrate competency	1	29	3%

*High‐volume training centers defined as performing >300 EUS/ERCPs per year.

Accordingly, the corresponding changes to the text appear below:

1. In the Results section of the Abstract, the sentence should have read:
  Competence is assessed in 65.9% of centers, but validated tools are applied in only 25.9%.
2. In the “Simulation‐based training” of the Results section, the affected sentences have been updated to:
  Although PD/Experts mentioned the availability of endoscopy simulators in 53.7% (*n* = 22) of departments, only 6 respondent trainees from 5 departments referred to have access to them during their training (…).
  Trainees use simulators at different stages of training: before (*n* = 10; 45.5%), at the beginning (*n* = 7; 31.8%), or during the whole hands‐on training period (*n* = 5; 22.7%).
3. In the “Competence assessment” of the Results section, the affected sentences have been updated to:
  Twenty‐seven departments (65.9%) perform a formal assessment during ERCP/EUS training programs, which is done: at set intervalst hroughout the fellowship (*n* = 12; 44.4%); randomly throughout the fellowship (*n* = 7; 25.9%); at the end of the fellowship (*n* = 8; 29.6%).
  The method(s) used to assess whether the trainee achieved endoscopic competence in ERCP/EUS are adequate performance on specific quality metrics, for example, cannulation rate or documentation of EUS landmarks (*n* = 15; 55.6%); the achievement of certain benchmarks (e.g., procedure volume) (*n* = 14; 51.9%); verbal attending evaluations (*n* = 11; 40.7%); adequate performance on a skills assessment tool, for example, The ERCP and EUS Skills Assessment Tool and Direct Observation of Procedural Skills (DOPS) (*n* = 7; 25.9%); written attending evaluation (*n* = 3; 11.1%).
4. In the “Discussion” section, the affected sentence should have read:
  “However, around 50% of departments do not have a formal ERCP/EUS curriculum and around one third do not perform any kind of formal assessment of trainee performance. Although validated assessment tools are formally used in only 25.9% of those who do, more than half of the training programs measure traditional benchmarks and performance metrics.

We apologize for these errors.

